# Supplementary material for: A Lipid Bodies-Associated Galactosyl Hydrolase Is Involved in Triacylglycerol Biosynthesis and Galactolipid Turnover in the Unicellular Green Alga Chlamydomonas reinhardtii
Source: Plants (Basel). 2021 Mar 31;10(4):675. doi: 10.3390/plants10040675 (PMC8065580; doi:10.3390/plants10040675)
Supplement: Supplementary file 1 [file plants-10-00675-s001.pdf]

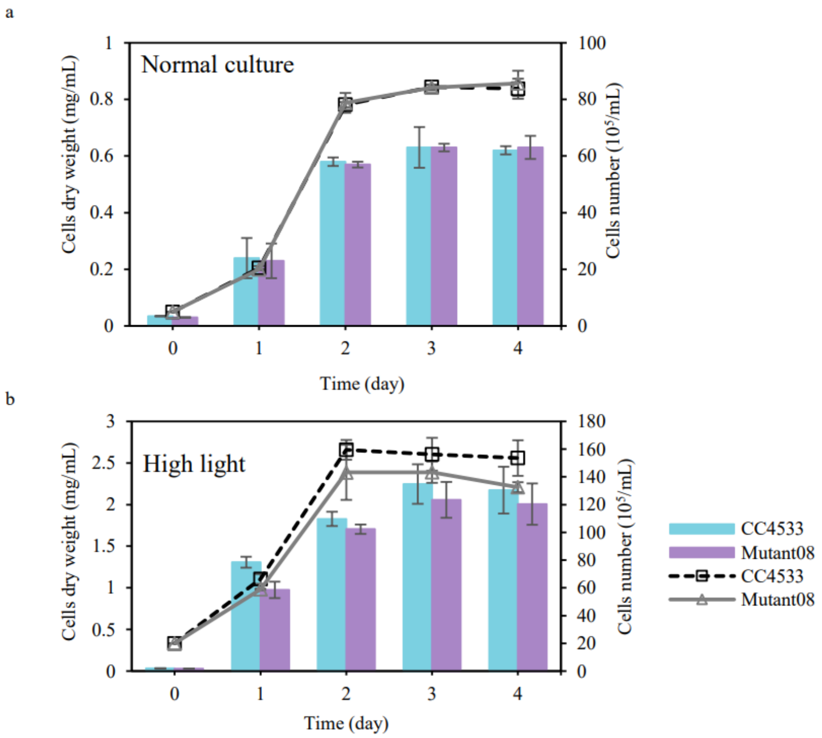

Figure S1. The growth rates of the *CrGH* insertion mutant and the wild type cc4533 strains grown under various culturing conditions. a and b represented the cell growth and dry weight of *C. reinhardtii* under the normal culture and high light stress conditions, respectively. The line chart represented the cells number and the histogram represented cells dry weight.

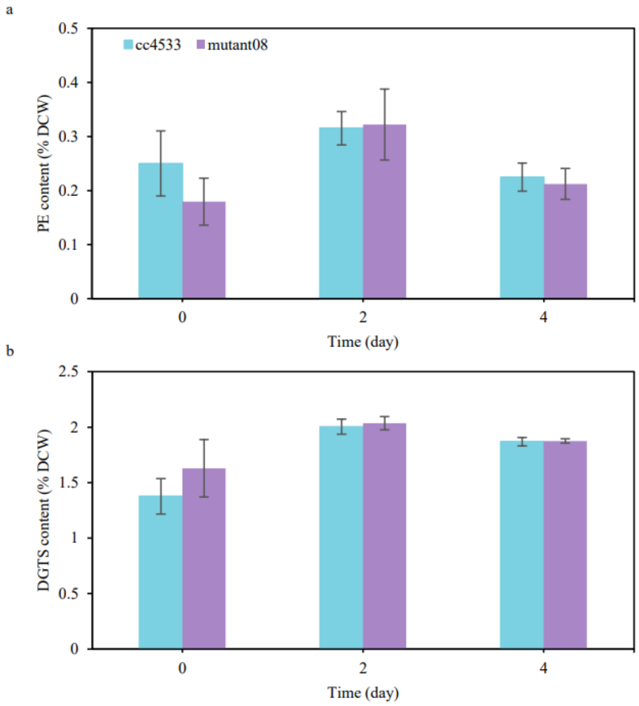

Figure S2. The extraplastidic membrane lipids contents in the *CrGH* insertion mutant and wild type cc4533. a. The PE contents. b. The DGTS contents. Data are presented as average ± SD (n = 3).

Table S1. Primers used for *CrGH* gene cloning and PCR analysis.

Primer Name.	Primer Sequences (5′ to 3′ end)...
CrGH-F.	TTAGAATTCATGACGGGCTCGGGCAAGCCCGCAGCGAAT...
CrGH-R.	TAACTCGAGTCATCCAATCGCCACCATGCCCACCGCCTT...
CrGH-mbp-linker-F.	TCAGGATCCGGAGGAGGTGGATCAGGAGGAGGTGGATCAGGA GGAGGTGGATCAATGACGGGCTCGGGCAAGCCCGCAGCGAAT...
CrGH-mbp-linker-R.	TCAAAGCTTTTAATGGTGATGGTGATGATGTCCAATCGCCACCA TGCCCACCGCCTT...
CrGH-qPCR-F.	ACTGGTGGGGCATCAACTACTAC...
CrGH-qPCR-R.	GTCTCAGTGATGTACATGGGAATG...
α-tubulin-F.	CTCGCTTCGCTTTGACGGTG...
α-tubulin-R.	CGTGGTACGCCTTCTCGGC...
Primer-G1.	TAAGGACAGCACAAGGGGAC...
Primer-C1.	GCACCAATCATGTCAAGCCT...
Primer-G2.	TCAAGGACAAGATCACGCTG...
Primer-C2.	GACGTTACAGCACACCCTTG...

Table S2. The subcellular location prediction of CrGH by WoLF PSORT.

Id	Site	Distance	Identity	Comments
At1g75950.1	cysk_nucl	190.02	10.0394%	[Arath]
CAT3_MAIZE	mito	191.935	10.8949%	[Uniprot] SWISS-PROT45: Mitochondrial.
At1g21100.1	cyto	199.102	12.9921%	[Arath]
IDHC_SOYBN	cyto	206.176	16.5029%	[Uniprot] SWISS-PROT45: Cytoplasmic.
At4g13940.1	mito	212.354	12.5245%	[Arath]
CYC_CUCMA	mito	214.152	7.08661%	[Uniprot] SWISS-PROT45: Mitochondrial matrix.
ENO_ALNGL	cyto	216.093	13.189%	[Uniprot] SWISS-PROT45: Cytoplasmic.
GLN2_MAIZE	cyto	217.781	16.1417%	[Uniprot] SWISS-PROT45: Cytoplasmic.
ALF_ORYSA	cyto	224.151	13.189%	[Uniprot] SWISS-PROT45: Cytoplasmic.
At1g20630.1	pero	227.252	10.1961%	[Arath]
EF2_BETVU	cyto	227.729	14.4721%	[Uniprot] SWISS-PROT45: Cytoplasmic.
ALF_MAIZE	cyto	229.056	12.9921%	[Uniprot] SWISS-PROT45: Cytoplasmic.
CYC_BRAOL	mito	229.404	7.28346%	[Uniprot] SWISS-PROT45: Mitochondrial matrix.
IPYR_ORYSA	cyto	241.248	10.2362%	[Uniprot] SWISS-PROT45: Cytoplasmic.
